# Transfusion-Dependent Anemia Secondary to Bowel Hemangiomas Managed With Sirolimus Therapy: A Case Report and Literature Review

**DOI:** 10.7759/cureus.107804

**Published:** 2026-04-27

**Authors:** Arush E Michael, Chirag Trivedi, Vijay Chaudhary, M Joseph John

**Affiliations:** 1 Clinical Hematology, Hemato-Oncology & Bone Marrow Transplant, Christian Medical College & Hospital, Ludhiana, IND

**Keywords:** anemia, gastrointestinal bleeding, intestinal hemangiomas, sirolimus, transfusion-dependent anemia

## Abstract

A real diagnostic challenge is posed by transfusion-dependent anemia in young patients with obscure gastrointestinal (GI) bleeding. Such a presentation can have many etiologies. Although rare, small intestinal hemangiomas are benign vascular tumors that frequently elude timely diagnosis owing to their intramural growth pattern and deep submucosal sequestration. Recent literature has increasingly shown the benefit of systemic therapies, such as sirolimus, for managing complex vascular anomalies. We report the case of a 17-year-old girl with a history of easy fatigability, melena, and exertional dyspnea, requiring regular red blood cell transfusions for the past six years. Initial laboratory investigations were suggestive of severe iron deficiency anemia, with a hemoglobin level of 6.3 g/dL and a ferritin level of 5.2 ng/mL. The stool occult blood test was positive. However, repeated upper and lower GI endoscopies and imaging were non-contributory. A repeat computed tomography angiography (CTA) of the abdomen was carried out, which showed intramural polypoidal lesions in the distal ileum and descending colon. Diagnostic laparoscopy was confirmatory of multiple small intestinal hemangiomas. In view of the multifocal nature of the lesions and the prohibitive risk of extensive bowel resection, a conservative strategy utilizing systemic mTOR (mechanistic target of rapamycin) inhibition was adopted. The patient achieved transfusion independence within two months, with a sustained normalization of hematological indices and a favorable side-effect profile. This case highlights the diagnostic complexity of small intestinal hemangiomas. It reinforces the role of imaging and laparoscopy, as well as the importance of considering rare causes, in transfusion-dependent anemia with chronic GI bleeding. In multifocal involvement of hemangiomas, sirolimus therapy may be an effective and safer alternative to surgical intervention. This case calls for more research and awareness of such rare vascular anomalies to accelerate diagnosis and management.

## Introduction

Gastrointestinal (GI) bleeding is a potentially life-threatening condition and may result from a wide range of underlying causes. An estimated 150 individuals per 100,000 population are admitted to hospitals annually in the United States and the United Kingdom due to GI bleeding, with a 5%-10% mortality rate. GI bleeding can be classified according to the rate of blood loss, visibility, and the source of the bleeding. It is classified as overt (acute), occult (chronic), or obscure GI bleeding. Overt or acute GI bleeding is visible to the naked eye and may present as hematemesis, melena, or hematochezia; whereas occult or chronic GI bleeding occurs due to microscopic hemorrhage and may present insidiously as heme-positive stools. Obscure GI bleeding may either be overt or occult and is defined as recurrent bleeding with an unidentifiable source [1]. There are many causes of obscure GI bleeding, and hemangiomas remain rare yet crucial differentials. Hemangioma of the small intestine is a rare disease, accounting for 7%-10% of all benign tumors of the small intestine [2]. These growths have no preference for either sex but typically occur in younger age groups [3]. In the small intestine, the jejunum is the most common site of occurrence for GI hemangiomas [4]. Obscure GI bleeding is the most common presenting symptom, but it may be accompanied by non-specific symptoms such as intestinal obstruction, abdominal pain, intramural hematoma, perforation, platelet sequestration, or intussusception [5]. When extensive or multiple, small intestinal hemangiomas can lead to chronic blood loss and anemia severe enough to require regular transfusions [6].

Standard diagnostic modalities, such as esophagogastroduodenoscopy, colonoscopy, and routine contrast-enhanced computed tomography, may be unsuccessful in identifying these lesions due to their submucosal location and often multifocal nature. This necessitates high clinical suspicion and the use of advanced imaging, and sometimes exploratory laparoscopy [7].

Surgical resection, which is relatively more invasive, is the conventional treatment modality for intestinal hemangiomas [8]. With the improvement of endoscopic therapeutic interventions, less invasive procedures are becoming increasingly employed. Systemic medical therapies, including sirolimus, an mTOR (mechanistic target of rapamycin) inhibitor, have gained attention, particularly in cases with diffuse involvement or high surgical risk. Sirolimus has antiangiogenic properties and is a promising therapeutic option in hemangiomas and other vascular anomalies [9]. We report a unique case of transfusion-dependent anemia in a young adult due to small intestinal hemangiomas, successfully managed with sirolimus. This case emphasizes diagnostic and therapeutic obstacles, underscoring the necessity for increased awareness of vascular anomalies in patients with unexplained GI bleeding and chronic anemia.

## Case presentation

Patient information

A 17-year-old female from Northern India presented to the hematology clinic with a protracted seven-year history of fatigability and exertional dyspnea. She also had a history of black-colored stool for the past six years. There was no history of recurrent jaundice, dysmenorrhea, menorrhagia, or bowel and bladder complaints. There is a medical history of hospitalization for similar complaints one year prior. There is a significant history of recurrent blood transfusions from a local government hospital. The patient exhibited profound transfusion dependency, necessitating approximately 25-30 units of packed red blood cells (PRBCs) over the preceding six years to maintain hemodynamic stability. The patient has a history of swellings developing in the hip and shoulder, five and 14 years before presentation, respectively. There was no medical or family history of malignancies or hemoglobinopathies.

Clinical findings

During general physical examination, she was found to have severe pallor and localized cystic swellings at the right shoulder and in the genital region. The abdomen was soft, non-tender, and bowel sounds were present. On per rectal examination, anal tone was normal, stool staining was noted, and there was no blood present on the examining finger. The liver was palpable 2 cm below the right costal margin, while the spleen was not palpable. There was no icterus, lymphadenopathy, clubbing, or edema.

Diagnostic assessment

Initial hematological profiling revealed profound microcytic hypochromic anemia, characterized by a hemoglobin level of 6.3 g/dL, a mean corpuscular volume (MCV) of 65.5 fL, and a mean corpuscular hemoglobin concentration of 30.8 g/dL. The anemia was regenerative as portrayed by the reticulocyte count of 9.9%. Total leukocyte and platelet counts remained within physiological reference ranges at 7,000/mm³ and 210,000/mm³, respectively. Biochemical analysis confirmed severe iron deficiency, evidenced by a markedly suppressed serum ferritin of 5.1 ng/mL (Table 1). Coagulation studies, including prothrombin time (PT), activated partial thromboplastin time (aPTT), and fibrinogen levels, were unremarkable, effectively ruling out systemic bleeding diathesis. Given the clinical history of melena, the presence of GI hemorrhage was objectively confirmed via a positive fecal occult blood test.

**Table 1:** Initial laboratory investigations suggestive of iron deficiency anemia Laboratory investigations are suggestive of iron deficiency anemia with low hemoglobin, microcytosis (low mean corpuscular volume, high red cell distribution width, low ferritin, and positive stool occult blood indicating possible chronic blood loss.

Parameters	Patient values	Reference range
Hemoglobin	6.3 g/dL	12-16 g/dL (female)
Total leukocyte count	7,000 cells/mm³	4,000-11,000 cells/mm³
Platelet count	210,000 cells/mm³	150,000-450,000 cells/mm³
Absolute neutrophil count	5,100 cells/mm³	1,500-8,000 cells/mm³
Mean corpuscular volume	65.5 fL	80-100 fL
Mean corpuscular hemoglobin concentration	30.8 g/dL	31.5-34.5 g/dL
Reticulocyte count	9.9%	0.3-1.0%
Red cell distribution width	24%	11.5-14.5%
Ferritin	5.2 ng/mL	15-200 ng/mL (female)
Stool occult blood	Positive	Negative

**Figure 1:**
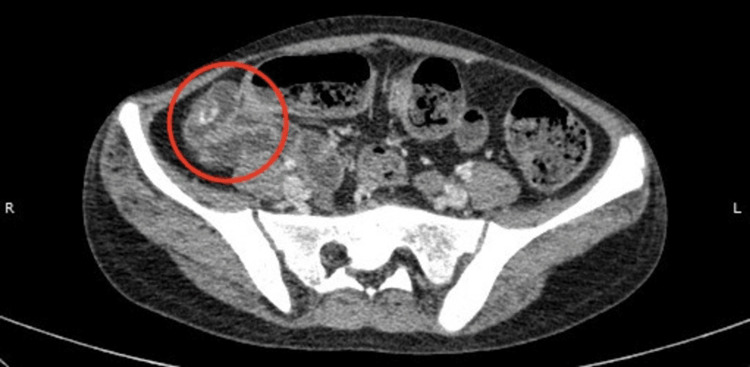
CTA of the abdomen showing bowel hemangiomas A computed tomography angiography (CTA) of the abdomen was planned. The red circle highlights a few intramural polypoidal lesions with intraluminal growth patterns involving the distal ileum and descending colon. These lesions show heterogeneous enhancement in the venous phase; a possibility of bowel hemangiomas was suggested

In view of the investigation findings, a provisional diagnosis of severe iron deficiency anemia and significant GI blood loss was made. For further evaluation, an upper GI endoscopy and colonoscopy were performed, which were inconclusive. Repeat endoscopy and colonoscopy were also normal. Ristocetin cofactor assay and testing for paroxysmal nocturnal hemoglobinuria were negative. A contrast-enhanced computed tomography (CECT) of the abdomen, which was done in an outside hospital, was normal. This further amplified the diagnostic dilemma of a transfusion-dependent anemia with GI blood loss with no obvious identifiable lesion. The differentials of Meckel's diverticulum and a small intestinal malignancy or angiovenous malformation were considered. A computed tomography angiography (CTA) of the abdomen was planned, and it showed a few intramural polypoidal lesions with intraluminal growth patterns involving the distal ileum and descending colon largest of which measures 14.2 mm (AP) x 17.7 mm (TR) x 13.6 mm (CC) in the distal ileum and 18.2 (AP) x 17.9 mm (TR) x 13.7 mm (CC) in the descending colon. These lesions show heterogeneous enhancement in the venous phase; a possibility of bowel hemangiomas was suggested (Figure 1). The CTA also showed multiple variable-sized soft tissue density lesions showing internal calcifications and heterogeneous enhancement in the left gluteus medius and right gluteus minimus, right gluteus maximus, and left tensor fascia lata. These soft tissue findings were suggestive of hemangiomas as well.

The patient was referred to the pediatric surgery team, where she underwent a diagnostic laparoscopy in which multiple hemangiomas were visualized alongside the length of the small bowel loops. The ileo-caecal junction and proximal small bowel were also visualised and were normal. Since no diverticulum or ectopic mucosa was visualised during the diagnostic laparoscopy, the diagnosis of Meckel's diverticulum was excluded intraoperatively. Given the CTA results showing distinct intramural polypoidal lesions with soft-tissue characteristics and the presence of comparable lesions in extraintestinal locales, arteriovenous malformations (AVMs), which usually manifest as flat or mild mucosal vascular lesions, were deemed less likely. In addition, the lesions' gross appearance and multifocal intramural nature during diagnostic laparoscopy were more consistent with hemangiomas than with AVMs [1]. Due to the lesions' multifocal distribution and the substantial risk of severe intraoperative bleeding, tissue biopsy was not attempted in this instance since the lesions were highly vascular. In addition, keeping in mind the possibility of significant morbidity, a major intestinal resection undertaken solely for diagnostic confirmation was deemed inappropriate. This extensive evaluation journey shaped up into a final diagnosis of transfusion-dependent anemia with recurrent GI blood loss secondary to multiple small intestinal hemangiomas.

Therapeutic intervention

The curative treatment for small intestinal hemangiomas is resection with end-to-end anastomosis. Considering the long-term morbidity and mortality associated with the surgical intervention, she was started on oral sirolimus and propranolol in August 2024. Iron supplementation was also initiated. Sirolimus and propranolol combination treatment was initiated for the patient. The sirolimus starting dose was one mg/day. To reach maintenance levels, the sirolimus dose was increased to two mg/day in two doses over a period of two months. With careful monitoring for bradycardia, hypotension, and hypoglycemia, propranolol was started at one mg/kg/day and subsequently raised to two mg/kg/day, over a period of three months. To address iron deficiency secondary to chronic GI bleeding, oral iron supplementation was also administered in the form of ferrous fumarate at 98.6 mg/day of elemental iron in a single dose.

Follow-up and outcomes

**Figure 2:**
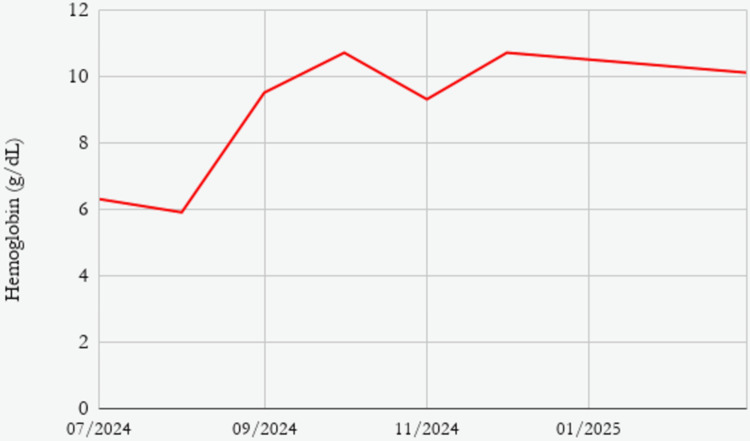
Line chart showing patients’ hemoglobin levels during clinical course and follow-up This line graph demonstrates the patient's haemoglobin (Hb) levels (g/dL) stabilising over the clinical course. The y-axis displays hemoglobin concentration, while the x-axis shows the timeline (July 2024 to March 2025). After initiating treatment, Hb levels increased significantly to 9.5 g/dL in September 2024 and 10.7 g/dL in October 2024 from 6.3 g/dL in July 2024. Overall improvement and stabilisation were shown by subsequent results that fluctuated little, with 9.3 g/dL in November 2024, 10.7 g/dL in December 2024, and 10.1 g/dL in March 2025.

**Figure 3:**
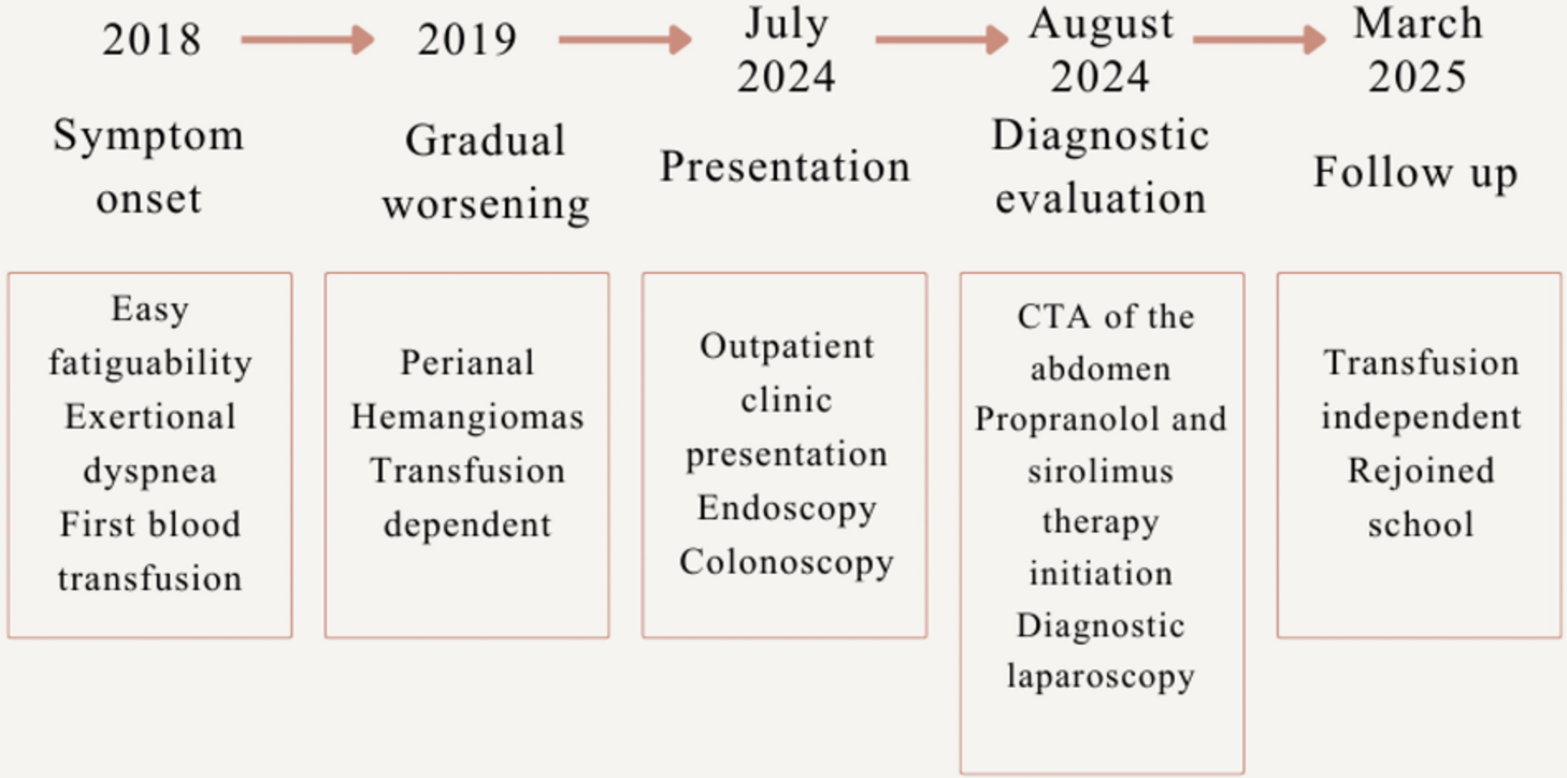
Timeline of the clinical course Clinical course timeline from symptom onset (2018) to follow-up (March 2025), highlighting progressive anemia, transfusion dependence, diagnostic evaluation and initiation of treatment. CTA: computed tomography angiography

Following the start of sirolimus therapy, the patient underwent frequent clinical and laboratory evaluations. Complete blood counts were performed periodically. To evaluate the treatment response, haemoglobin levels and transfusion requirements were monitored. Over the course of two months after initiation of therapy, her hemoglobin improved to 10.7 g/dL and remained stable thereafter (Figure 2). Her symptoms subsided, and she has remained transfusion independent over the past six months. Clinical surveillance focused on assessing signs of infection, mucositis, and other drug-related adverse effects, including oral ulcers, peripheral edema, and delayed wound healing. No sirolimus-related adverse events were observed. She had been unable to attend school over the past four years due to her symptoms; she has now rejoined school. A timeline of the patient’s clinical course from symptom onset to follow-up has been outlined in Figure 3. While serial radiological imaging to assess lesion regression was not performed, a repeat diagnostic laparoscopy at 9 months demonstrated persistence of small bowel hemangiomas. Although this confirms ongoing structural disease, the patient showed significant clinical improvement with sustained transfusion independence. In view of persistent lesions on follow-up, combination therapy with sirolimus and propranolol was continued.

Patient perspective

The patient expressed relief at achieving transfusion independence and reported significant improvement in quality of life, allowing her to resume school after several years of interruption.

Informed consent

Written informed consent was obtained from the patient for the publication of this case report.

## Discussion

Small intestinal hemangiomas are uncommon vascular lesions that rarely present in adults and can pose diagnostic dilemmas when causing obscure GI bleeding. Intestinal hemangiomas originate from the submucosal vascular plexus and involve the mucosa, muscularis, and mesentery. Hemangiomas are histologically classified as capillary hemangioma, cavernous hemangioma, and mixed hemangioma [10]. These benign tumors may remain undiagnosed for years, especially when located in the jejunum or ileum [3]. This literature review involved searching the PubMed database for papers published in the last 15 years regarding GI hemangiomas. The search terms utilized included “hemangioma”, “gastrointestinal bleeding”, “chronic anemia”, “vascular malformation”, or “oral propranolol”, which were used in various combinations to find the most suitable studies for this review.

A manual search was also performed using the references of eligible articles. The exclusion criteria were non-English papers. A total of 11 original papers were identified and reviewed in Table 3 (11 case reports). There were eight females and three males; the youngest patient was three months old, while the oldest patient was 81 years old. Patients presented with occult or overt GI bleeding (five cases), anemia and its symptoms (six cases), abdominal discomfort (six cases), and, in severe cases, repeated transfusion (one case), as observed in our patient. The diagnostic yield of conventional endoscopy is often limited by the deep submucosal sequestration of these lesions, warranting the early integration of capsule endoscopy (CE) or balloon-assisted enteroscopy (BAE). CE remains a pivotal tool for initial visualization, while BAE allows for tissue sampling and therapeutic interventions. Endoscopy techniques were used to diagnose eight cases. Out of the 11 cases, CE and/or BAE were used to diagnose five cases, and exploratory laparoscopy was used for the diagnosis of one case. In our case, a CECT of the abdomen was used to suggest the possibility of a bowel hemangioma, and further localisation was performed using an exploratory laparoscopy. The literature review underscores a persistent diagnostic latency, where conventional endoscopic modalities frequently lack the sensitivity to detect intramural vascular proliferations, necessitating the early adoption of deep enteroscopy or cross-sectional enterography. When these modalities confirm the presence of vascular malformations, further evaluation is required to determine the extent of the lesion and decide on management.

**Table 2:** Literature review of bowel hemangioma cases with clinical and treatment profiles CE: capsule endoscopy; BAE: balloon-assisted enteroscopy; MRI: magnetic resonance imaging

Author	Year of publication	Age	Sex	Presenting symptoms	Diagnostic modality	Location	Type of hemangioma	Treatment and outcome
Durer et al. [3]	2018	66 years	Male	Worsening leg pain	CE, BAE	Jejunum	Cavernous	Not detailed
Al-Tkrit A et al. [4]	2020	32 years	Male	Syncope and dizziness	Exploratory laparoscopy	Jejunum	Cavernous	Surgical resection/good outcome
Coleman et al. [6]	2018	2 years	Female	Recurrent episodes of severe anemia	Technetium‑99m pertechnetate scintigraphy	Small bowel	Not specified	Surgical resection/good outcome
Hu PF et al. [7]	2018	24 years	Female	Recurrent melena and fatigue for one year	CE, BAE	Ileum	Cavernous	Surgical resection/good outcome
Numan et al. [8]	2020	81 years	Female	Melena	Push enteroscopy	Jejunum	Not specified	Surgical resection/good outcome
Wei Y et al. [10]	2023	56 years	Male	Hematochezia for one day	BAE	Jejunum	Cavernous	Surgical resection/good outcome
Vlad RM et al. [11]	2024	6 years and 6 months	Female	Severe anemia and repeated blood transfusions	Exploratory laparoscopy	Jejunum and ileum	Not specified	Surgical resection/good outcome
Helal et al. [12]	2024	3 months	Female	Rectal bleeding and paroxysmal crying	Exploratory laparotomy	Jejunum	Mixed	Surgical resection/good outcome
Francesca Ré et al. [13]	2024	79 years	Female	Melena for one month	CE	Jejunum and ileum	Cavernous	Surgical resection/good condition but experiencing diarrhea
Kleinman et al. [14]	2023	3 months	Female	Pallor, restlessness around feeding, and failure to gain weight for three weeks	Abdominal MRI	Diffuse small bowel	Not specified	Oral sirolimus and propranolol/good outcome
Katsurahara et al. [15]	2023	68 years	Female	Anemia	CE, BAE	Jejunum	Cavernous	Surgical resection/good outcome

In the differential diagnosis, syndromic causes of vascular abnormalities were taken into account, although the overall clinical picture deemed them improbable. In addition to internal organ involvement, these diseases frequently exhibit identifiable external indicators that aid in diagnosis. An array of systemic features, such as characteristic cutaneous vascular lesions, limb asymmetry or hypertrophy, and extensive venous or lymphatic abnormalities, are typically present in disorders including blue rubber bleb nevus syndrome and Klippel-Trénaunay syndrome. Therefore, rather than a broader congenital vascular syndrome, clinical findings in this case are more consistent with isolated, non-syndromic multifocal hemangiomas [16].

Traditionally, localized lesions have been managed with surgical resection, offering a definitive cure [7]. However, in cases of multifocal or diffuse hemangiomas, surgery may not be feasible. Complete resection of the widely distributed lesions along the small intestine can often require extensive or multiple segmental resections, predisposing patients to short bowel syndrome, malabsorption, and significant postoperative morbidity. In addition, it is possible to miss an occult lesion during surgery, which may potentially result in persistent bleeding.

Emerging evidence supports the use of systemic therapy with sirolimus, an mTOR inhibitor with antiangiogenic and antiproliferative properties, particularly in syndromic or refractory cases [14]. Sirolimus has demonstrated efficacy in reducing lesion size and controlling bleeding in various vascular anomalies and has been increasingly used in pediatric and adult populations. In our patient, sirolimus therapy led to a marked reduction in transfusion requirements and stabilization of hemoglobin levels. The clinical response to sirolimus in this patient is mechanistically plausible, as sirolimus binds intracellular FKBP12 and the resulting complex inhibits mTORC1, a key signaling node governing cellular growth, protein synthesis, and proliferation [17]. Given that many vascular anomalies are driven by dysregulation of the PI3K-AKT-mTOR axis, pharmacologic mTOR inhibition provides a rational targeted strategy to suppress endothelial proliferation and pathologic angiogenesis [18]. Sirolimus inhibits angiogenesis by suppressing HIF-1α-dependent vascular endothelial growth factor (VEGF) expression, thereby reducing endothelial proliferation and abnormal vessel formation [19]. In multifocal intestinal hemangiomas, this may translate into reduced lesion activity and vessel friability, thereby limiting chronic GI blood loss and allowing correction of transfusion-dependent anemia. No major adverse effects were observed during follow-up, and the patient remained clinically stable. This case highlights the importance of considering small intestinal hemangiomas in the differential diagnosis of transfusion-dependent anemia and the promising role of sirolimus as a non-surgical therapeutic option.

Limitations

This is a case report describing a single patient with a brief follow-up and little generalisability. The non-availability of advanced small intestinal assessment techniques, such as CE and device-assisted enteroscopy, which might have improved lesion localisation and characterisation, was one of the limitations encountered. Another limitation of this report is the absence of intraoperative photographic documentation, as intraoperative images were not captured during the diagnostic laparoscopy. Although sirolimus dosing was adjusted clinically, complete serial trough level monitoring data were not available, limiting objective pharmacokinetic correlation. The use of propranolol and sirolimus combination treatment makes it challenging to delineate the individual contribution of each agent. 

## Conclusions

Bowel hemangiomas remain a rare but significant source of obscure GI bleeding and transfusion-dependent anemia. This case emphasizes the significance of maintaining a high index of suspicion in patients with prolonged unexplained anaemia. Employing multimodal diagnostic techniques, such as advanced imaging and laparoscopy, may help reach a diagnosis. When resection is not feasible, non-surgical management with agents such as sirolimus may offer an alternative in multifocal hemangiomas. Future prospective studies are essential to develop standardised treatment procedures and assess the long-term safety and effectiveness of sirolimus-based therapy in vascular malformations, as the evidence is still limited.
